# Giant Panda (*Ailuropoda melanoleuca*) Buccal Mucosa Tissue as a Source of Multipotent Progenitor Cells

**DOI:** 10.1371/journal.pone.0138840

**Published:** 2015-09-23

**Authors:** Hilary M. A. Prescott, Craig Manning, Aaron Gardner, William A. Ritchie, Romain Pizzi, Simon Girling, Iain Valentine, Chengdong Wang, Colin A. B. Jahoda

**Affiliations:** 1 Durham University, School of Biological and Biomedical Sciences, Durham, DH1 3LE, United Kingdom; 2 Roslin Embryology Ltd., 21 St Germains Terrace, Macmerry, East Lothian, EH33 1QB, United Kingdom; 3 Royal Zoological Society of Scotland, Corstorphine Road, Edinburgh, EH13 6TS, United Kingdom; 4 China Conservation and Research Centre for Giant Panda (CCRCGP), Shi Qiao Village, Qing Chenshan Town, DuJiangYan City, SiChuan Province, 611844, China; University of Newcastle upon Tyne, UNITED KINGDOM

## Abstract

Since the first mammal was cloned, the idea of using this technique to help endangered species has aroused considerable interest. However, several issues limit this possibility, including the relatively low success rate at every stage of the cloning process, and the dearth of usable tissues from these rare animals. iPS cells have been produced from cells from a number of rare mammalian species and this is the method of choice for strategies to improve cloning efficiency and create new gametes by directed differentiation. Nevertheless information about other stem cell/progenitor capabilities of cells from endangered species could prove important for future conservation approaches and adds to the knowledge base about cellular material that can be extremely limited. Multipotent progenitor cells, termed skin-derived precursor (SKP) cells, can be isolated directly from mammalian skin dermis, and human cheek tissue has also been shown to be a good source of SKP-like cells. Recently we showed that structures identical to SKPs termed m-SKPs could be obtained from monolayer/ two dimensional (2D) skin fibroblast cultures. Here we aimed to isolate m-SKPs from cultured cells of three endangered species; giant panda (*Ailuropoda melanoleuca*); red panda (*Ailurus fulgens*); and Asiatic lion (*Panthera leo persica*). m-SKP-like spheres were formed from the giant panda buccal mucosa fibroblasts; whereas dermal fibroblast (DF) cells cultured from abdominal skin of the other two species were unable to generate spheres. Under specific differentiation culture conditions giant panda spheres expressed neural, Schwann, adipogenic and osteogenic cell markers. Furthermore, these buccal mucosa derived spheres were shown to maintain expression of SKP markers: nestin, versican, fibronectin, and P75 and switch on expression of the stem cell marker ABCG2. These results demonstrate that giant panda cheek skin can be a useful source of m-SKP multipotent progenitors. At present lack of sample numbers means that we can only postulate why we were unable to obtain m-SKPs from the lion and red panda cultures. However the giant panda observations point to the value of archiving cells from rare species, and the possibilities for later progenitor cell derivation.

## Introduction

Skin-derived precursor (SKP) cells are a population of multipotent progenitor cells, which possess the ability to differentiate along various lineages including neural crest and mesenchymal progeny [1]. Multipotent progenitor cells are considered to be a valuable resource for regenerative medicine; firstly because they are often easier to access than more potent cell types and secondly as it is often possible to isolate patient specific populations, avoiding issues arising from the use of allografts. Originally isolated from skin, SKPs have also been sourced from human hair follicle and murine vibrissa dermal papilla cells [2–4]. Although previous reports suggest that SKP numbers decline with age [5], a more recent report has shown that SKPs can efficiently be obtained from the cheek/chin tissue of aged individuals [6]. SKPs are conventionally obtained from freshly isolated tissue, but we have previously demonstrated that identical structures, termed m-SKPs, could also be derived from monolayer human skin fibroblast cultures including those that had been previously cryopreserved [7]. Surprisingly, because the hair follicle dermal papilla has previously been identified as an enriched niche for SKPs [2–4], m-SKPs could not be derived from cultured human hair follicle dermal papilla cells in the same study. Therefore suggesting that the body sites and tissues from which SKPs and m-SKPs can be produced are not entirely predictable.

Up to now SKPs have only been obtained from humans and domestic and laboratory animals. Given the interest in using cellular manipulation to preserve rare and endangered mammals [8,9], and the relative absence of information about how readily stem cells can be readily obtained from these animals, we set out to determine whether m-SKPs could be established from three different mammalian species. Here we demonstrate that m-SKPs could be isolated from cultured buccal mucosa fibroblasts from giant panda (*Ailuropoda melanoleuca*) but that skin dermal fibroblasts cultured from red panda (*Ailurus fulgens*) and Asiatic lion (*Panthera leo persica*) were not able to produce m-SKPs.

## Materials and Methods

### Ethics

Work was approved by the Royal Zoological Society of Scotland (RZSS) ethics and welfare committee and Durham University Life Sciences Ethical Review Process (LSERP) Committee. The Red panda and Asiatic lion were euthanased on welfare grounds by the Royal Zoological Society of Scotland (RZSS) zoo veterinarians. Both animals were anaesthetised with medetomidine and ketamine administered intramuscularly by carbon dioxide powered dartgun, then euthanased by administration of pentobarbitone intravenously in the jugular vein. Giant pandas were anaesthetised as approved by the RZSS ethics and welfare committee for semen collection and artificial insemination with medetomidine and ketamine administered intramuscularly by carbon dioxide powered dartgun, followed by endotracheal entubation and anaesthesia maintenance with sevoflurane in oxygen. Approval for the culture work in Durham was given by the Durham University Life Sciences Ethical Review Process (LSERP) Committee. Project title: “Culture of cells and derivation of stem cells from zoo/captive wild animal tissues.”

### Isolation and culture of buccal mucosa and skin dermal fibroblasts (DF)

Biopsy specimens were obtained from the buccal mucosa of two anaesthetised giant pandas (one male and one female) and from the abdominal skin of a red panda and an Asiatic lion, both recently deceased. The buccal mucosal samples were small pieces of surplus tissue from biopsies taken to assess the genetic health of the individual pandas, the health and welfare of the pandas’ potential offspring, and giant panda population health in captivity. The tissue was sterilised using 10% Videne followed by successive washes in 70% ethanol and sterile PBS. Biopsies were finely minced and placed in a culture flask with modified Eagle’s medium (MEM) (Life Technologies—42360–081), 10% foetal bovine serum (FBS-Sigma—F7524), 100units/ml penicillin and 100μg/ml streptomycin (Life Technologies—15070063) and 0.5μg/ml amphotericin B (Life Technologies—15290018). Fibroblastic cells were cultured in a 37°C incubator with 5% CO2 and media was changed every 3–4 days. Cells were cryopreserved in 90% FBS and 10% DMSO (Sigma—D2650).

### Formation and culture of m-SKPs

The culture of m-SKPs was performed using the method described by Hill *et al*. [7]. In all cases monolayer cell cultures between passages 2 and 5 were enzymatically passaged at 80% confluence using 0.25% Trypsin-EDTA (Life Technologies—25200–072) and re-seeded in SKP proliferation media. This consisted of a 3:1 ratio mixture of DMEM (Life Technologies—10567–014):F12 (Life Technologies—31765–035), 40 ng/ml of basic Fibroblast Growth Factor (Peprotech—100-18B), 20 ng/ml of Epidermal Growth Factor (Peptrotech—AF-100-15) and 2% v/v of B27 (Life Technologies—17504044). m-SKP cultures were supplemented with fresh 10 x SKP proliferation media every 3–4 days. Images were captured with a Zeiss Axiovert 10 microscope using Axiovision software.

### IHF analysis of m-SKPs

m-SKPs were snap frozen in optimal cutting temperature (OCT) compound (Fisher- LAMB/OCT) and sectioned at 8μm. Sections were either fixed in 4% paraformaldehyde (PFA) for 1 hour at room temperature and permeabilised with 0.1% Triton X-100, or fixed in ice-cold acetone for 30 minutes, and blocked with 3% bovine serum albumin (BSA) (Sigma-A2153). Primary antibodies were added in PBS containing 3% BSA and left at 4°C overnight. The following primary antibodies were used: Versican (1:10—DSHB—12C5), Fibronectin (1:100—Sigma—F3648), nestin (1:100—Millipore—MAB5326), βIII Tubulin (1:100—Sigma—T8660), S100β (1:100—Sigma—S2532) and α-SMA (1:100—Abcam—ab7817). Specimens were then washed in PBS before incubation with appropriate fluorophore conjugated secondary anti mouse IgG (Life Technologies—A11029) or anti rabbit IgG (Life Technologies—A11012) antibodies. Nuclei were stained with 4', 6-diamidino-2-phenylindole (DAPI) prior to mounting with Mowiol. Images were captured using Zeiss imager M1 fluorescent microscope with Velocity software (Improvision).

### Differentiation of m-SKPs

m-SKPs were dissociated as described above and plated in SKP adherence media overnight. SKP adherence media consisted of 1x SKP proliferation media supplemented with 10% FBS. Cells for neuronal and Schwann differentiation were seeded on 0.02 mg/ml laminin (Sigma—L4544) and 0.2 mg/ml poly-D-lysine (Sigma—P7280) coated glass coverslips in SKP adherence media. Adhered SKP cell cultures were supplemented with either neuronal, Schwann cell, osteogenic or adipogenic differentiation media (S1 Table) when confluent. Fresh differentiation media was added every 3–4 days. Cells supplemented with osteogenic or adipogenic media were cultured for 14 days or 28 days for cultures supplemented with neuronal and Schwann cell differentiation media.

### Analysis of monolayer SKP cultures

Cells cultured in osteogenic and adipogenic differentiation media were analysed using Von Kossa staining and Oil Red-O staining respectively as previously described [7]. Immunofluorescent analysis was performed for cultures supplemented with neuronal and Schwann cell differentiation media using βIII tubulin or S100β antibodies respectively, as previously described [10].

## Results

**Fig 1:**
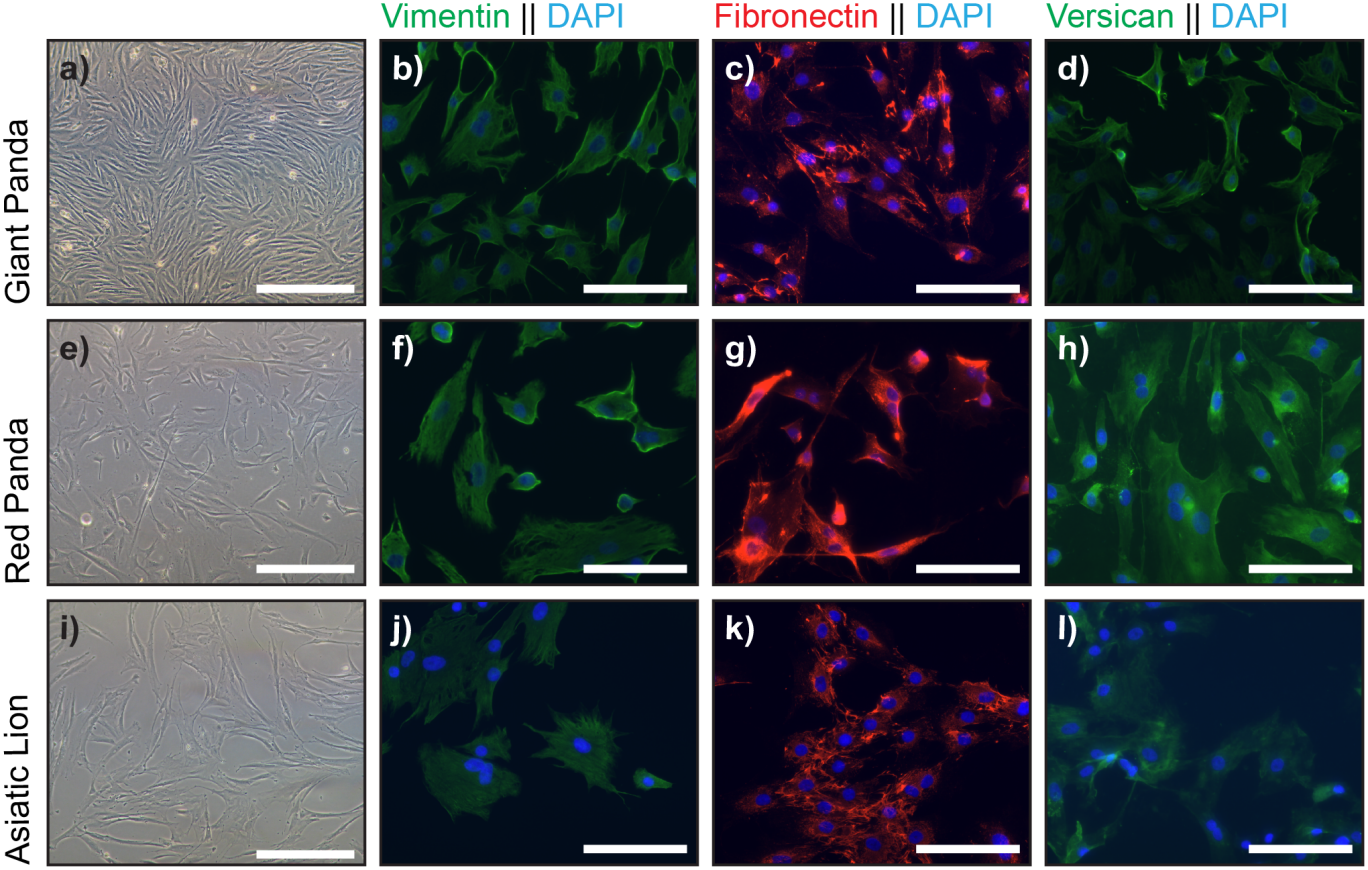
Monolayer fibroblasts cell cultures express markers associated with SKPs. DF cells isolated from skin biopsies from giant panda (**a**), red panda (**e**) and Asiatic lion (**i**) could be cultured as monolayers. Giant panda (**b-d**), red panda (**f-h**) and Asiatic lion (**j-l**) monolayer DF cultures all expressed vimentin, fibronectin and versican. Nuclei were stained with DAPI. Scale Bar = 100μm.

**Fig 2:**
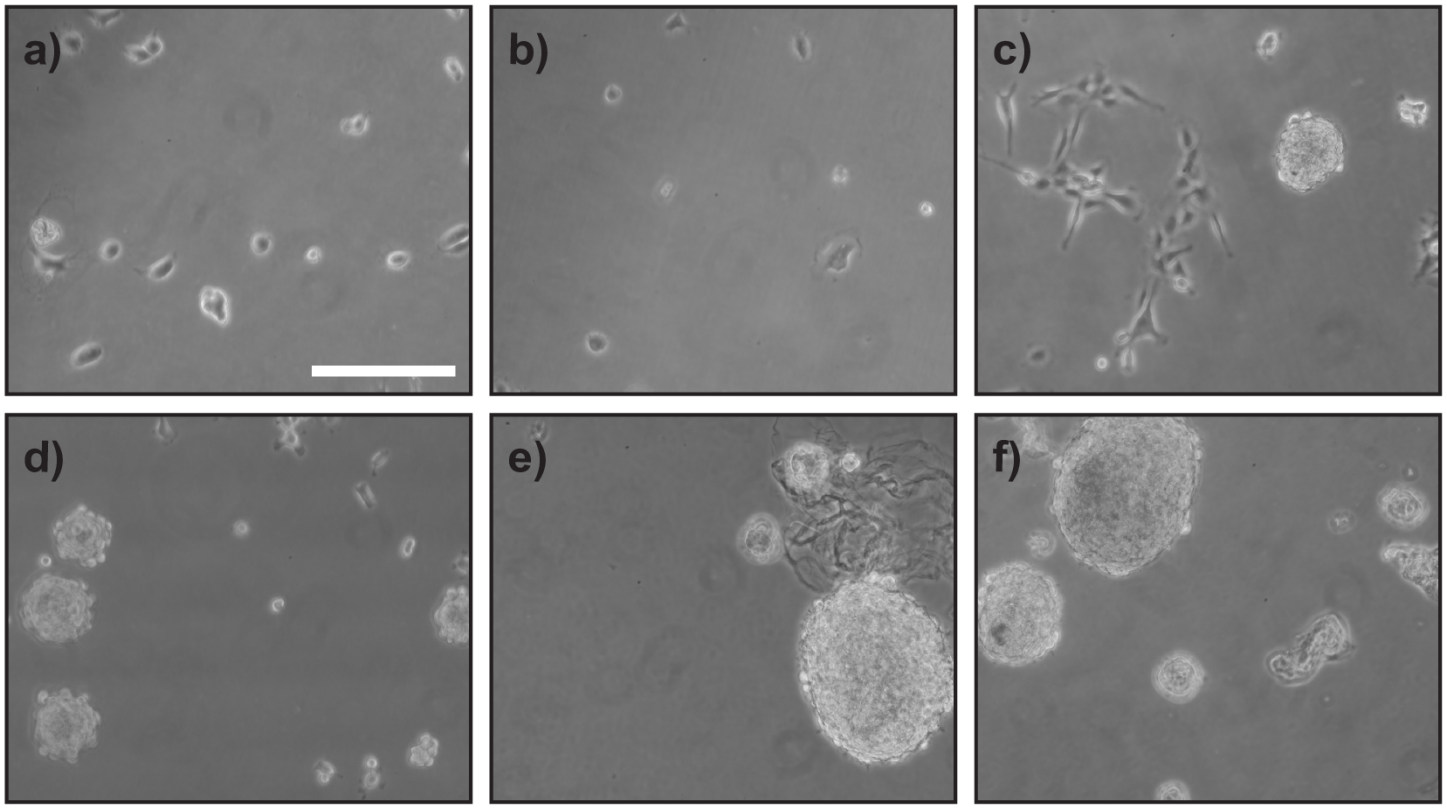
Giant panda fibroblasts, but not red panda or Asiatic lion fibroblasts, are able to generate m-SKPs after passage and cryopreservation. Fibroblast cultures from red panda (**a**) and Asiatic lion (**b**) were unable to generate m-SKPs. Female giant panda fibroblasts cultures were able to generate m-SKPs (**c**). Buccal mucosa cell cultures from the male giant panda could also generate m-SKPs (**d**) after cryopreservation of cells, m-SKPs generated from the male giant panda could be passaged to p1 (**e**) and p2 (**f**). Scale Bar = 100μm.

Cultures were successfully initiated from all three sources of tissue, with fibroblast-like cells predominant in the explant outgrowths, and universal in all the subcultured populations (Fig 1). All three showed positive immunofluorescent staining with vimentin antibody, confirming their mesenchymal character. They also all labelled positively with antibodies to the extracellular matrix molecules, fibronectin and versican (Fig 1). Although dermal fibroblast cells isolated from human skin from various body sites, including abdominal skin, have previously been shown to be capable of generating SKPs and m-SKPs [7,11], we were unable to generate m-SKP-like spheroids when fibroblasts cultured from the abdominal tissue of the red panda and Asiatic lion were put into SKP medium (Fig 2). However, m-SKP-like spheroids were obtained from cultured male and female giant panda buccal mucosa fibroblasts over multiple passages (p2 to p5) (Fig 2). The number of spheroids was relatively low, ranging from 29 to 108 per 25cm2 flask at P2, their size also varied. However, we were also able to generate m-SKP-like spheroids from buccal mucosa fibroblast cultures after cryopreservation. Moreover, we found that male giant panda spheroids could themselves be passaged/split at least twice (Fig 2).

**Fig 3:**
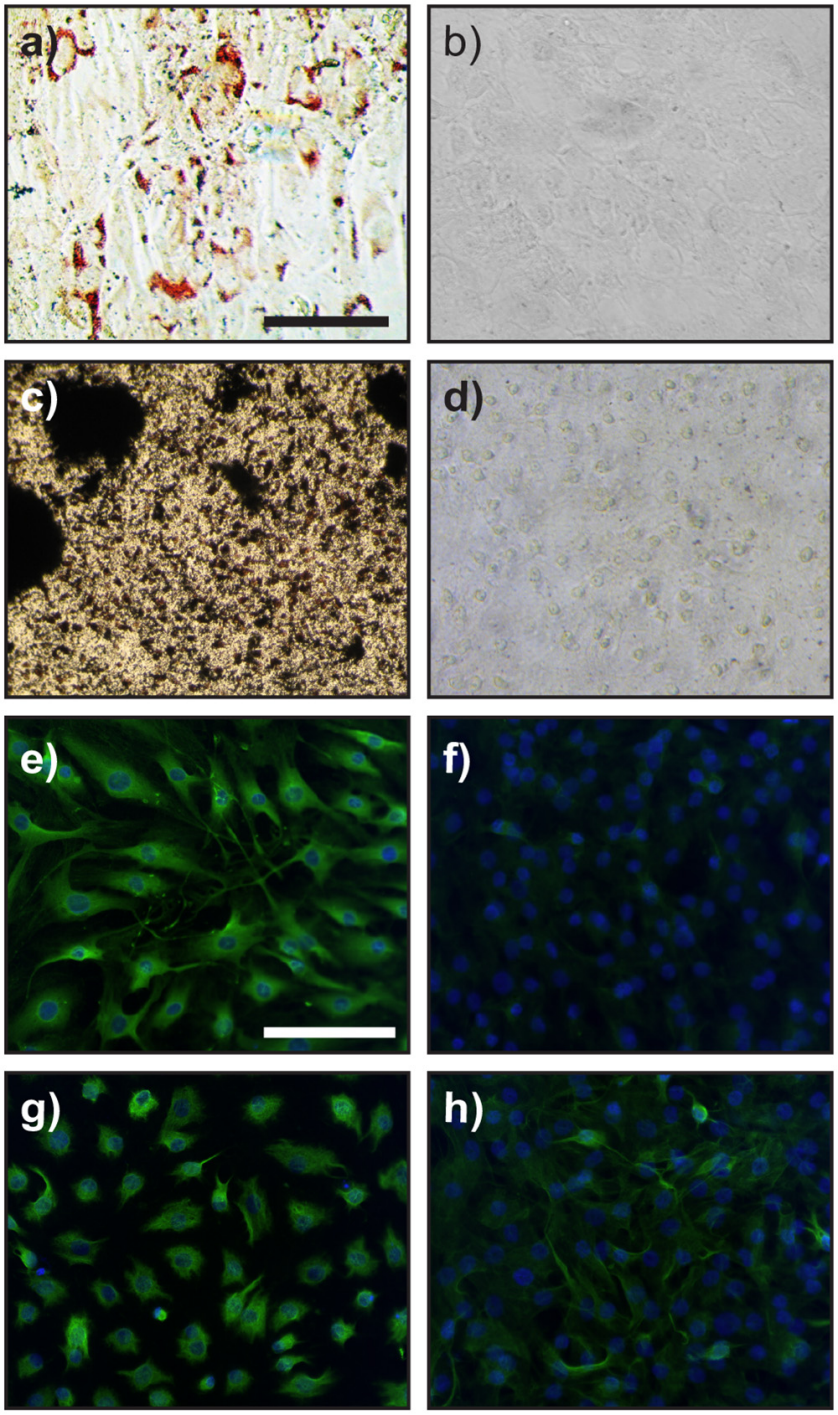
Giant panda buccal mucosa m-SKPs are able to differentiate along various cell lineages. Giant panda cells from passage 3 m-SKPs cultured in adipogenic and osteogenic differentiation media exhibited adipogenic differentiation (**a**) and osteogenic differentiation (**c**) as shown by oil Red-O and Von Kossa staining, respectively. Control cultures (**b**) and (**d**) did not stain positively for adipogenic or osteogenic differentiation, respectively. Giant panda m-SKPs cultured in neuronal differentiation media differentiated along neuronal lineages as shown by βIII tubulin immunostaining (**e**). βIII tubulin staining was negative in SKP cells cultured in control media (**f**). The Schwann cell marker S100β was up-regulated in cells cultured in Schwann cell differentiation media and cell morphology was characteristic of Schwann cells (**g**) when compared to cells cultured in control media (**h**). Nuclei were stained with DAPI in (**e-h**). Scale Bar = 100μm.

To test whether the giant panda-derived spheroids had the multipotent properties of m-SKPs, they were reintroduced into 2D culture in various differentiation media. m-SKP cells from both the first round of production, and those that had been passaged twice were tested for osteogenic and adipogenic differentiation capabilities by culturing in specific medium for 14 days. Cells were then stained, and positive differentiation was confirmed using Von Kossa and Oil Red-O staining respectively (Fig 3). Negative controls cultured in MEM with 10% FBS did not exhibit calcification or lipid deposition (Fig 3). After 28 days in neuronal and Schwann cell medium, first production m-SKP cells showed evidence of positive differentiation, confirmed using immunofluorescence staining with neuronal and Schwann cell markers: βIII tubulin or S100β respectively. Control cultures supplemented with MEM with 10% FBS did not label positively for βIII tubulin but some did label positively for S100β, although to a lesser extent than that observed in differentiated cultures (Fig 3). In addition, the morphology of cells cultured with Schwann cell medium was more consistent with that of isolated Schwann cells.

**Fig 4:**
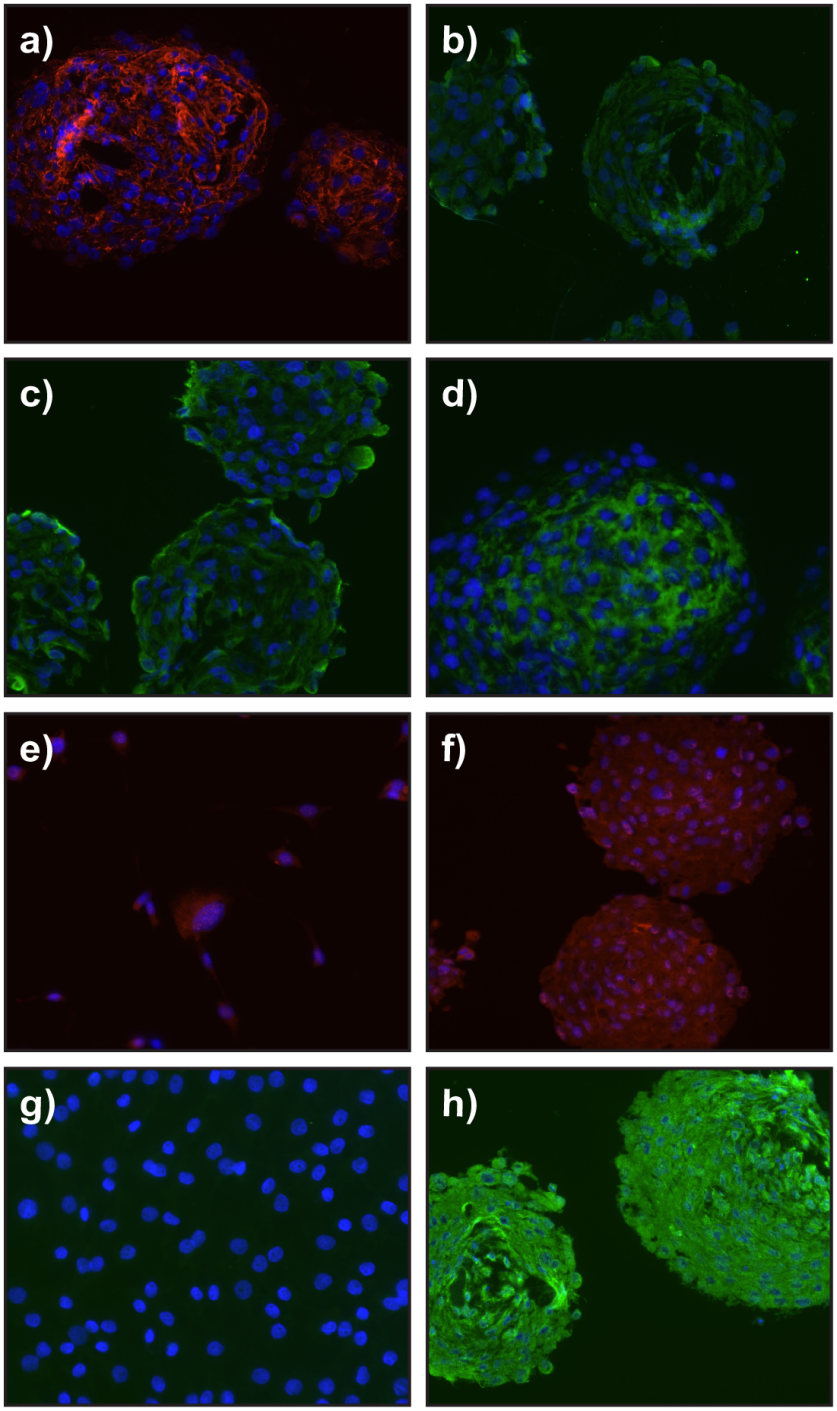
Expression of SKP protein markers is maintained and ABCG2 expression is up-regulated in giant panda m-SKPs. Expression of SKP associated markers was analysed in m-SKPs generated and passaged from giant panda male buccal mucosa cell cultures. Fibronectin (**a**), nestin (**b**), vimentin (**c**), and versican (**d**) are expressed in m-SKPs. P75 similarly is expressed both in monolayer (**e**) and m-SKP (**f**) cells. ABCG2 expression is not expressed in giant panda monolayer cultures (**g**) but is induced in m-SKPs (h). Nuclei were stained with DAPI. Scale bar = 100μm.

**Fig 5:**
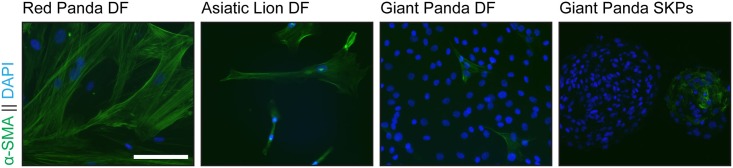
m-SKP yield does not correlate with α-SMA expression in red panda, Asiatic lion or giant panda buccal mucosa fibroblasts. Monolayer cultures of red panda (**a**) and Asiatic lion (**b**) dermal fibroblasts strongly expressed α-SMA, compared to monolayer cultures of giant panda buccal mucosa fibroblasts (**c**) where α-SMA expression was greatly reduced. Some, but not all giant panda SKP spheroids displayed α-SMA expression (**d**). Nuclei were stained with DAPI. Scale Bar = 100μm.

Giant panda buccal mucosa spheres were then examined and shown to express five SKP markers, fibronectin, nestin, versican, vimentin and P75 (Fig 4). However as there was overlapping expression with previous observations in monolayer culture (Fig 1) expression of ABCG2, a broad marker of stem cells in a wide variety of tissues [12], was also investigated. In monolayer giant panda DF cultures ABCG2 was not expressed; however, upon m-SKP formation high levels of ABCG2 expression were observed. Both red panda and Asiatic lion dermal fibroblasts expressed high levels of alpha smooth muscle actin relative to the giant panda cells (Fig 5).

## Discussion

Since the discovery of SKPs, their potential for regenerative medicine has progressed considerably [13–15]. However as a readily available source of adult stem/progenitor cells SKPs could be important in other domains, including conservation of endangered species. All these possibilities are further enhanced by the discovery that m-SKPs could be derived from cultured cells [7,16]. Here we showed that m-SKP-like spheroids could be obtained from cultured giant panda cells over multiple passages. It was not possible to use the full molecular toolkit to confirm that the spheroids being produced were SKPs as sequence information on some of the key panda genes is not available. However at the protein level in cases where antibodies were effective, all the relevant markers were positive. Crucially, ABCG2 which is a good indicator of stem cells and is expressed in SKPs [12,17] was switched on only in those cells that had formed spheroids. These data, combined with the capacity of the spheroid cells to differentiate down multiple lineages, including after the m-SKPs had been multiply passaged, strongly suggested that m-SKPs had been produced. As we showed previously with cultured human skin fibroblasts [7] m-SKPs from the giant panda could be produced after monolayer cultures had been cryopreserved.

At present we are unable to say why the giant panda cells and not those from the red panda or Asiatic lion were able to make m-SKPs. In all cases the first attempt to derive SKPs was made at relatively early passage number. One obvious explanation, is the body site difference, since the buccal cavity has recently been shown to be a good source of SKPs [6]. Indeed the buccal mucosa is generally a privileged area for wound healing [18] and may therefore be a site rich in progenitor cells Nevertheless m-SKPs have been obtained from cultured fibroblasts from human abdominal skin. Working on human skin fibroblast cultures we previously observed a correlation between high α-SMA expression and greater m-SKP formation [7]. However, despite showing high levels of α-SMA (Fig 5), both red panda and Asiatic lion dermal fibroblasts were unable to generate m-SKPs. Conversely, giant panda buccal mucosa cells showed low levels of α-SMA. Therefore with these three cell populations there was no correlation between high levels of α-SMA expression and m-SKP formation. Another difference is that the giant panda cells were obtained from living animals while the skin samples from the red panda and lion were from animals that had been euthanised because of age/ill health. In humans a link has been made between the age of individuals from whom skin has been taken and the capacity of the skin to generate SKPs [5]. Ultimately, however, more work will be needed to support this or any other explanation with any degree of confidence.

The use of nuclear transfer based cloning to preserve endangered species has already been pioneered using the oocytes from a closely related domestic species, for example with the mouflon [19]. Moreover, induced pluripotent stem cells (iPSCs) have been created from somatic cells of the northern white rhinoceros [20] and of the snow leopard [21] establishing a platform for future reintroduction of genetic material into the breeding pool either “as a source of reprogrammed donor cells for nuclear transfer or for directed differentiation to gametes” [21]. Recent work has demonstrated that by generating induced pluripotent stem cells it was possible to improve the efficacy of somatic cell nuclear transfer (SCNT) [22], a precursor step in interspecies cloning, which may also be of use in preserving endangered species. Indeed, up to the present iPS technology has proven to be readily achievable for a wide range of species, and would currently be the method of choice for these strategies. As yet, however, no giant panda iPS cells have been produced. Although, SKPs have not yet been shown to be advantageous in the context of cloning, they are potentially interesting candidates for this approach, and we hypothesise that use of culture-derived progenitors like m-SKPs could provide an increase in cloning efficiency, whilst avoiding any potentially harmful effects of iPS cells produced with viral vectors. More generally, because of the precarious situation of endangered species, it is important that when dealing with available material we arm ourselves with all of the knowledge and as many of the tools in the toolbox, using whatever samples we can collect at an opportunistic time, whether that be in the field or in captivity. Therefore it is worthwhile obtaining any new information/knowledge about the stem/progenitor capabilities of existing cells. As m-SKPs are derived from cultured cells they have the advantage of being widely available from archived and banked cryofrozen cell culture collections. This could be of particular importance in the conservation field where efforts are already being made to culture and store cells from zoo animals for potential future study and application, via the “Frozen Zoo” [23] and “Frozen Ark” [24] projects.

## Supporting Information

S1 Table Summary of media constituents. (DOCX)
