# Supplementary material for: Giant Panda (Ailuropoda melanoleuca) Buccal Mucosa Tissue as a Source of Multipotent Progenitor Cells
Source: PLoS One. 2015 Sep 23;10(9):e0138840. doi: 10.1371/journal.pone.0138840 (PMC4580591; doi:10.1371/journal.pone.0138840)
Supplement: S1 Table — (DOCX) [file pone.0138840.s001.docx]

#### S1 Table - Summary of media constituents

| **Reagent** | **Concentration** | **Supplier** | **Catalogue Number** |
| --- | --- | --- | --- |
| **Adipogenic Differentiation Media - MEM** | | | |
| 3-isobutyl-1-methylxanthine (IBMX) | 0.45 nM | Sigma | I7018 |
| Insulin | 2.07 µM | Sigma | I3536 |
| Dexamethasone | 100 nM | Sigma | D4902 |
| Rabbit Serum | 15% v/v | Sigma | R4505 |
| Amphotericin B | 0.5 µg/ml | Life Technologies | 15290018 |
| Pen/Strep | 100 units/100 µg/ml | Life Technologies | 15070063 |
| **Osteogenic Differentiation Media – MEM** | | | |
| Dexamethasone | 100 nM | Sigma | D4902 |
| β-glycerophosphate | 10 mM | Sigma | G9422 |
| L-Ascorbic acid 2-phosphate | 50 µM | Sigma | A8960 |
| FBS | 10% v/v | Sigma | F7524 |
| Amphotericin B | 0.5 µg/ml | Life Technologies | 15290018 |
| Pen/Strep | 100 units/100 µg/ml | Life Technologies | 15070063 |
| **Schwann Cell Differentiation Media - DMEM:F12 (3:1)** | | | |
| Forskolin | 5 µM | Sigma | F3917 |
| Heregulin-1β | 50 ng/ml | Peprotech | 100-03 |
| N2 Supplement | 2 % v/v | Life Technologies | 17502 |
| FBS | 1 % v/v | Sigma | F7524 |
| **Neural Cell Differentiation Media - DMEM:F12 (3:1)** | | | |
| Neurotrophiin-3 | 10 ng/ml | Peprotech | 450-03 |
| Nerve Growth Factor | 50 ng/ml | Peprotech | 450-01 |
| Brain-Derived Neurotrophic Factor | 50 ng/ml | Peprotech | 450-02 |
| FBS | 1 % v/v | Sigma | F7524 |
